# Vinyl-Asbestos Floor Risk Exposure in Three Different Simulations

**DOI:** 10.3390/ijerph18042073

**Published:** 2021-02-20

**Authors:** Lorena Zichella, Fiorenza Baudana, Giovanna Zanetti, Paola Marini

**Affiliations:** Department of Environment, Land and Infrastructure Engineering (DIATI), Politecnico di Torino, 10129 Turin, Italy; fiorenza.baudana@polito.it (F.B.); giovanna.zanetti@polito.it (G.Z.); paola.marini@polito.it (P.M.)

**Keywords:** chrysotile asbestos, SEM analysis, vinyl-asbestos flooring, free asbestos fibers, simulation test

## Abstract

Vinyl floors are widely used in public areas for their low cost and easy cleaning. From 1960 to 1980, asbestos was often added to improve vinyl floor performances. The Italian Ministerial Decree (M.D.) 06/09/94 indicates asbestos vinyl tiles as non-friable materials and, therefore, few dangerous to human health. This work aims to check through three different experimental tests if asbestos floor tiles, after decades of use, maintain their characteristics of compactness and non-friability. The effect of a small stone fragment stuck in the sole of rubber shoes was reproduced by striking the vinyl floor with a crampon. A vinyl tile was broken into smaller pieces with the aid of pliers to simulate what normally happens when workers replace the floors or sample it to verify the presence of asbestos. The third test reproduced the abrasion of the tile surface due to the dragging of furniture or heavy materials or sand grains that remain attached to the soles of shoes. The tests were carried out in safe conditions, working under an extractor hood with a glove box. Airborne sampling in the hood obtained the concentration of asbestos fibers produced in each test. The simulation tests performed confirms the possible release of fibers if the vinyl tiles are cut, abraded or perforated, as indicated by the Italian M.D.

## 1. Introduction

Between 1960 and 1980, vinylasbestos was widely used for flooring public buildings, schools, hospitals and housing due to its mechanical characteristics, low cost, rapid installation and easy cleaning. The production of vinyl-asbestos flooring was carried outby mixing inert fillers, PVC (Polyvinyl chloride) resins, copolymers, pigments and large quantities of asbestos. The mixture was heated until ~150 °C to reach the desired plasticity and pressed to obtain the required thickness; it was then cut into tiles. The result is a coating very similar to linoleum. Asbestos added to the aggregates and PVC improved the properties of mechanical, heat and corrosion resistance.

**Figure 1:**
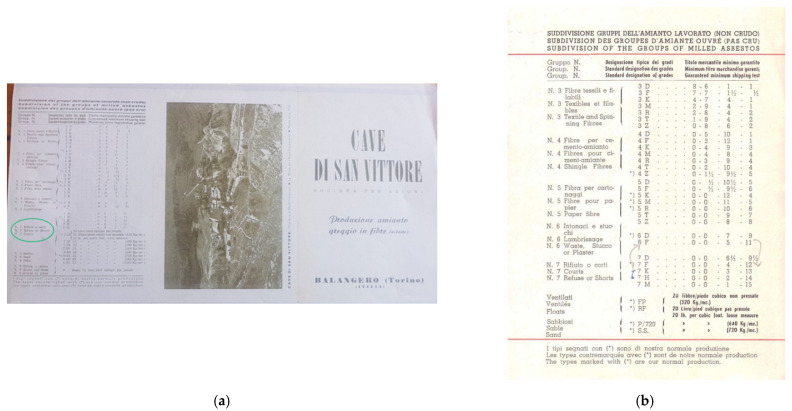
(**a**) San Vittore (Balangero, Turin, Italy) quarry, flyer extract for asbestos fiber production. Asbestos group 7 is circled in green, probably used for vinyl-asbestos floor. (**b**) Particularitiesof the subdivision of the group of milled asbestos (source: https://it.wikipedia.org/wiki/Amiantifera_di_Balangero; accessed on 10 January 2021).

From documents kept at the DIATI (Department of Environmental and Infrastructure Engineering) asbestos laboratory of the Politecnico di Torino, the asbestos fibers used for the production of vinylasbestos, produced in the San Vittore (Balangero, Piedmont, Italy) asbestos mine, as it is shown in the flyer of the mine products (Figure 1) were of Grade 7 [1].The length of the asbestos fibers of Grade 7 were <10 mesh (corresponding to <2 mm) and this was the typology of asbestos fiber with the lowest economic value. Among all the different types of asbestos of industrial use (Actinolite, Amosite, Anthophyllite, Chrysotile, Crocidolite, Tremolite, according to the experience in the DIATI asbestos laboratory of the Politecnico di Torino, after more than 500 analyses on vinyl asbestos samples, the only one asbestos detected in vinyl tiles was Chrysotile.

In the literature, there are several patents concerning the manufacture of vinyl-asbestos floors, two of which are given asexamples (see Table 1 and Table 2).

In addition, according to the US patent of Petry [2], asbestos particle parts by weight of the components and dimensions of fibrous particles are shown in Table 1. The specific weight of this vinyl-asbestos has values of 1.85–1.90 g/m^3^ but can reach 1.50 g/m^3^ depending on the type of filler used.

The percentage of components in the US patents of Laurito and Wheeler [3] is shown in Table 2.

The Italian Ministerial Decree (M.D.) 6/9/94 [4] established, in implementation of Article 6 of Law 257/92 [5], the methods for the removal of products containing asbestos, as well as those for transportation, storage of asbestos waste in landfills for special and hazardous waste, treatment, packaging and covering of materials containing asbestos. Furthermore, the procedures to be adopted for asbestos remediation are defined. The three remediation methods indicated by the Decree are the following:

- Removal—it is mandatory for brittle materials and compact materials in the case of damages for more than 10% of its surface.
- Encapsulation—covering of the asbestos-containing materials (ACMs) with several layers of a specific encapsulation coating, producing a physical barrier between the contaminated matter and the external environment.
- Over coverage—occulting and sealing the ACMs by means of the physical barrier such as panels, walls or insulation.

**Figure 2:**
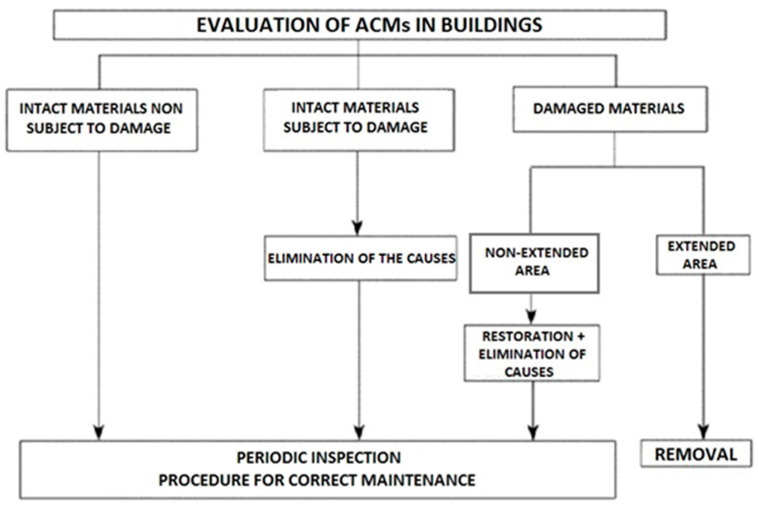
Evaluation scheme of asbestos-containing materials (ACMs) in building and recommendations (from Article 1, Annex: Standards and Technical Methods for Risk Assessment, Control, Maintenance and Remediation of Materials Containing Asbestos Present in Building Structure, Risk Assessment, Table 2 of M.D. 6/9/94 [4].

Figure 2 describes the different situations that can occur during the evaluation of ACMs in buildings and the related recommendations in these different cases required by M.D. 6/9/94 [4].

The Decree [4] also defines the criteria for choosing asbestos protection devices to be used by operators during the reclamation and methods of sampling and analysis of materials and environment to verify the presence of asbestos fibers.

In the case of encapsulation of ACMs, procedures and methods for rehabilitation and a control program maintenance are recommended instead of removal. This maintenance schedule must be periodic [4,6].

The mapping of asbestos is compulsory for every kind of asbestos material in civil buildings; the first step is to respect the evaluation of the risk and the program of management and custodial control [4,7,8].

Finally, the Lgs.D.9 April 2008 n. 81 [9] on hygiene and safety in the workplace and subsequent modifications Lgs. D. 3 August 2009 n. 106 [10] identify that the employer must ascertain the presence of ACMs. The employer must assess the risk of workers and act on this. Legislative Decree 81/2008 [9] defines the exposition limits value of 0.1 fiber per air cm^3^.

The ACM potential danger depends on possible fibers released in the air, which can be inhaled by occupants. The most important criterion to evaluate is the friability of the materials. Depending on the friability [4], ACMs can be classified in two categories. Friable ACMs can be easily crumbled or reduced to powder by the pressure of an ordinary human hand (this category includes non-friable asbestos materials where the matrix deteriorates due to weathering, ageing or wear). Non-friable ACMs are hard materials that can be crumbled or reduced to powder only with the use of mechanical tools (abrasive discs, cutter, drills, etc.); these are no longer able to maintain the original compactness. Once released, asbestos fibers are light enough to hang in the air for hours and days, long after other dust has settled; thus, being able to be inhaled. M.D. 6/9/94 [4] provides exposition limits aimed at protecting people from breathing airborne fibers. In Table 3 [4], indications on asbestos content and friability of the main building materials are given.

According to M.D. 6/9/94 [4], asbestos vinyl flooring contains chrysotile asbestos in the percentages from 10 to 25% (the same value of the US patents of Laurito and Wheeler [3], (Table 2)), and has an improbable release of fibers during normal use, but the possibility of released fibers if cut, abraded or perforated.

This is confirmed in the research of Campopiano et al. [11], on the problem of indoor asbestos in school buildings, where the sampling of airborne carried out over many years always showed a very low concentration of the asbestos fibers in asbestos vinyl tiles. Williams and Crossman [1] investigated the release of fibers during the removal of vinyl-asbestos flooring using the RCFI (Resilient Floor Covering Institute) recommendations [12] and noted that the release of the fibers does not occur when breaking tiles, but an important part of the asbestos is caused by abrasion between fragments of tiles and mastic and/or scraper.

Kominsky et al. [13] evaluated the release of fibers on three conditions of vinyl flooring (poor, medium and good) during spray buffering and wet stripping. The authors demonstrated that the high level of fibers released during this maintenance on the floor in bad condition was due to the decay of the matrix englobing the asbestos fibers.

Boulanger et al. [14] studied the presence of SAF (short asbestos fibers L < 5 micron) in air samples as an indicator of the degradation of ACM to identify pollution and anticipate health risk. Dodson et al. [15] have asserted in their research that asbestos fiber length is related to potential pathogenicity. The Regulation does not consider this type of fiber as dangerous to health, so in this research it is not taken into account.

Eypert-Blaison et al. [16] have shown that the notion of friable/non-friable for the Regulation was erroneous and unreliable insofar as the dust emitted by a given material depends not only on its nature but also on the removal technique and the collective protection means associated with it during work.

Perez et al. [17] evaluated the exposure to airborne asbestos during work on floor tiles, by means of a meta-analysis of the data collected through personal sampling on worker. They found that activities that involved polishing or bluing, scoring or snapping and scraping or lifting had the highest personal phase contrast optical microscopy (PCOM) concentrations, while floor tile removal and chemical solvent removal had the lowest concentrations. However, generally these works do not produce airborne concentrations at levels that exceed the current OSHA PEL [18].

According to a study conducted by Lange [19], it was found that during the demolition of floors and mastics containing chrysotile, there is a low exposure to airborne asbestos.

The purpose of the following research is to compare two different vinyl tiles containing asbestos. A first step provides vinyl tile characterization through density, macroscopic and microscopic analysis. A second step involves three different simulation tests to obtain useful guidelines for operators who remove or carry out maintenance.

## 2. Materials and Methods

### 2.1. Vinyl-Asbestos Characterization

**Figure 3:**
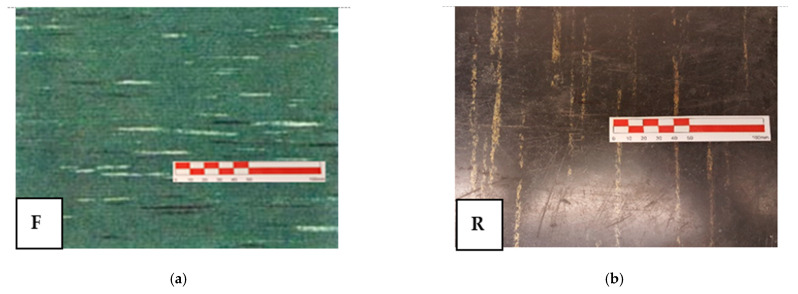
(**a**) Sample F—squared floor green tiles with white and black stripes, more flexible and in good condition (**b**) Sample R—squared floor black tiles with beige stripes, quite rigid and in fairly good condition.

The prevalent use of vinyl floors containing asbestos was in public buildings and popular housing. In this context, two types of vinyl from public buildings have been considered. In Figure 3, they are shown macroscopically. The green vinyl (F) on the left was laid in the 1990s and shows a flexible structure. The black vinyl (R) on the right was laid in the 1980s and shows a more rigid structure.

The density of the two vinyl samples, in line with patent [2], was calculated using surface/volume ratio. The results obtained are shown in Table 4.

The evaluation of the presence of asbestos was carried out with two methodologies: a simple abrasion of the surface material utilizing a scalpel; a sample burning at 500 °C of a tile fragment followed by a reaction with HCl, to concentrate the asbestos fibers which are resistant to this temperature.

The quantity and dimension of the asbestos (Chrysotile) are different in the two vinyl tiles. Concerning the quantity evaluation, it is difficult due to a non-homogeneous distribution of Chrysotile in the vinyl tiles. For this reason, the quantity defined by M.D. 6/9/94 [4] (10–25%) has been considered. The dimensions were observed through SEM/EDS (scanning electron microscope/energy dispersion micro analytical system) analysis, and the spectrum identification was carried out following the International Standard ISO 14966 [20].

**Figure 4:**
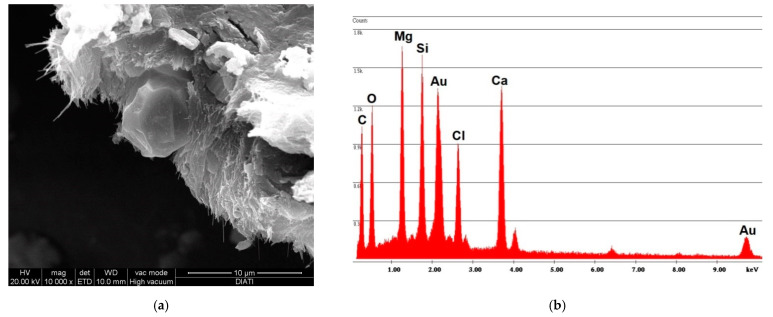
F sample SEM analysis. (**a**) Photo of incorporated fibers of chrysotile; (**b**) spectrum of chrysotile and vinyl chloride composition.

The simple abrasion of surface tile powder was analyzed with SEM/EDS; Figure 4 shows the spectrum obtained by analyzing the powder from vinyl F. The photo on the left shows how the fibers are completely incorporated within the vinyl matrix. The spectrum shows both of the elements that compose chrysotile, Mg and Si, where the Mg peak is greater than the Si peak; the spectrum also reports the elements that compose the vinyl matrix, Cl and Ca.

**Figure 5:**
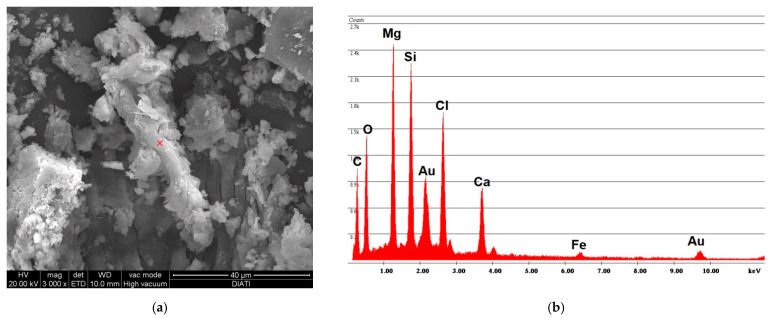
R sample SEM analysis. (**a**) Photo of incorporated fibers of chrysotile; (**b**) spectrum of chrysotile and vinyl chloride composition.

Figure 5 shows the same analysis, performed on the powder obtained from the vinyl sample R. In addition, in this case, the fibers are incorporated in the vinyl matrix; the spectrum confirms this aspect due to the presence of Mg and Si components of the chrysotile.

One feature that distinguishes asbestos, and which led to wide use in the past, is its stability at high temperatures. To obtain a higher concentration of asbestos sample from the vinyl, freeing the fibers, a burning treatment on a plate at 500 °C was carried out.

This procedure was used to better identify the fibers and to be able to measure them by SEM. This procedure is not possible with the simple scratching of the surface, as the fibers, in this case, remain incorporated in the matrix.

**Figure 6:**
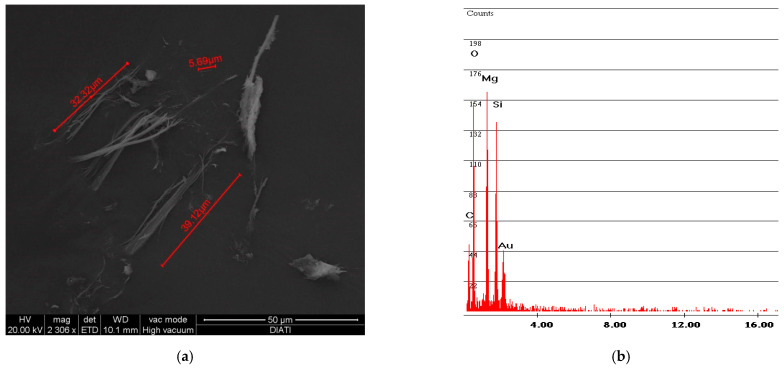
F sample SEM analysis. (**a**) Photo of free fibers of chrysotile with measurement values; (**b**) spectrum of chrysotile.

**Figure 7:**
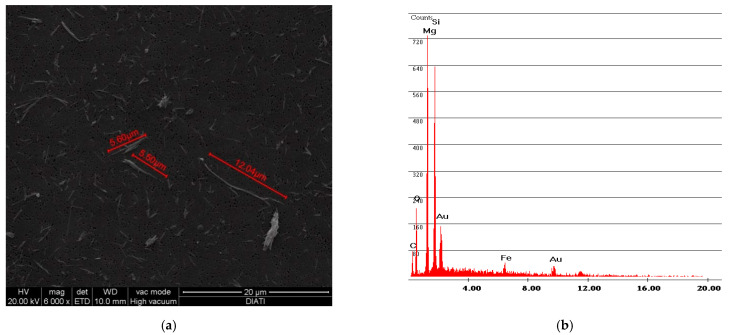
R sample SEM analysis. (**a**) Photo of free fibers of chrysotile with measurement values; (**b**) spectrum of chrysotile.

Figure 6 and Figure 7 show the free fibers obtained, respectively, from samples F and R. The measurements of the fibers of both samples are in line with the particle size dimension declared by Petry US Patent [2], that is, below 2 mm. A difference can also be observed between the particle size dimension of sample R and sample F: the older and more rigid sample R has smaller fiber sizes than sample F.

### 2.2. Simulation Tests

From Table 3 [4], for vinyl floors the fiber-release risk during normal use is low, but it is probable in the case of cutting, perforation or wear. To verify the release of fibers, the R and F samples were subjected to several simulation tests. For each simulation test, an evaluation of fiber release was carried out through the SEM/EDS analysis of airborne samples [20].

The three simulation tests were carried out all in dry conditions and are the following:

− Multigrip plier breakage to simulate when workers break the vinyl floor tiles while replacing them or sampling to verify the presence of asbestos.
− Impact and drag simulation with crampon; the effect of a small stone fragment stuck in the sole of rubber shoes was reproduced by striking the vinyl floor with a crampon.
− Abrasion using two different sandpaper grain sizes to simulate the smoothing of a floor where the portion of asbestos vinyl tiles sometimes rub together with glue remaining on the surface.

**Figure 8:**
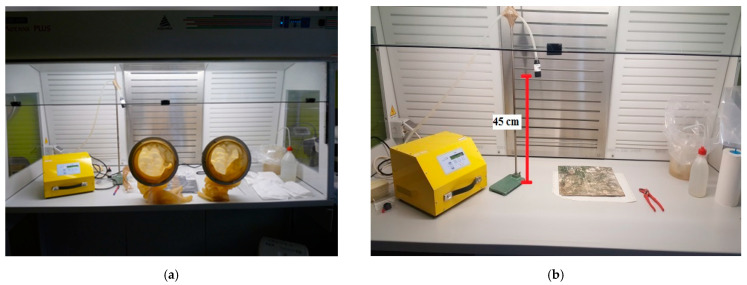
(**a**) Confined hood with gloves for simulation tests. (**b**) The airborne retrieval through a polycarbonate membrane.

Figure 8 shows the preparation of the hood to carry out the tests. All tests were performed safely in a confined environment using a glove box with the fan off. Airborne sampling was done with a polycarbonate membrane at 45 cm from the table. Between tests, a cleaning of the hood and an airborne analysis was always been carried out to verify the cleanness of the hood to avoid contamination.

All airborne sampling after cleaning and during the tests were carried out with flow rate 8 L/min and a sampling time of 180 min to obtain a volume of 1440 L.

The airborne samples were then analyzed with a SEM/EDS. The analyses were conducted at 3000X, examining 166 fields corresponding to 1.1 mm^2^. A phase-contrast microscope (PCOM) analysis of the powder obtained during abrasion was performed to verify the presence of free fibers.

#### 2.2.1. Sampling Simulation with Multigrip Plier Breakage in Dry Condition of Pieces of Vinyl Tile

**Figure 9:**
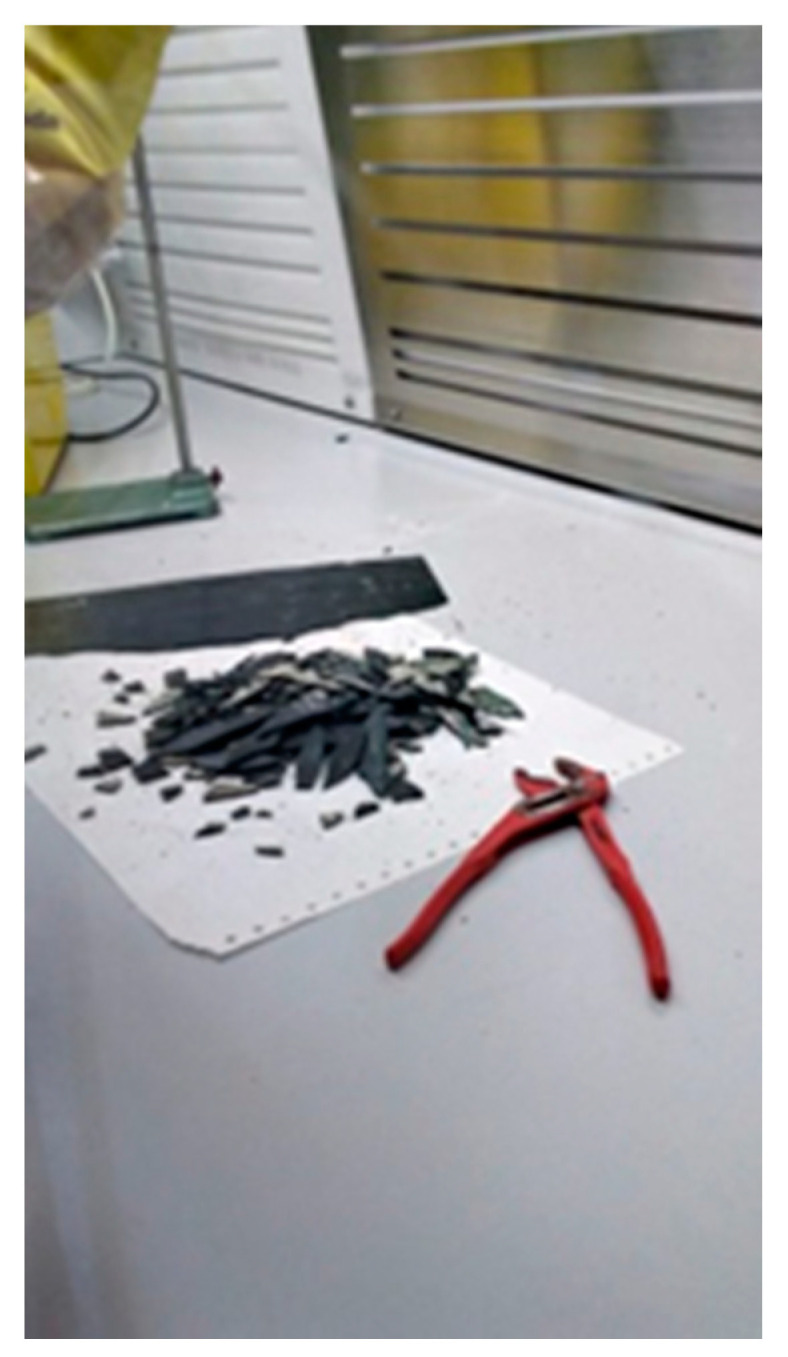
Multigrip plier breakage test.

During the sampling phase for the ACM mapping, a piece of tile is detached manually. This procedure must normally be carried out wet. The purpose of this test, however, was to check whether there is a release of fibers during this ACM sampling phase. In this case, the test was carried out in a confined environment, with multigrip pliers in dry condition (Figure 9). The duration time of the test was 15′. The airborne samples were then analyzed with SEM/EDS.

#### 2.2.2. Impact and Drag Simulation with Crampon in Dry Conditions

**Figure 10:**
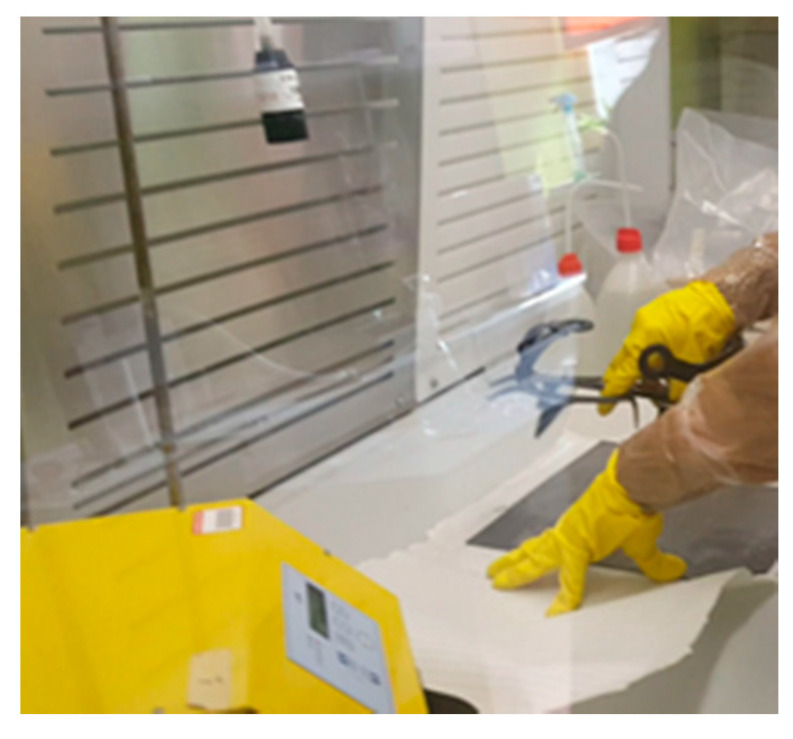
Vinyl tile crampon test inside the confined hood.

A vinyl floor is subject to pedestrian traffic. This test was carried out using NiCrMo steel crampons (Camp_Cassin brand (Premana, Italy)) with Hardness HRC (Rockwell scale) 53 ÷ 58, to simulate the pedestrian traffic with impact and drag. The test was performed with 100 crampon shots (impact and drag) by the same operator in ten minutes. This test was performed manually from about 40 cm in height by letting the crampon fall without applying force. The number of shots could underestimate the wear carried out over time by the pedestrian traffic, but the hardness of steel is greater than any shoe worn, therefore, a possible release of fibers during the test can be representative. Figure 10 shows the performed test.

#### 2.2.3. Abrasion Simulation Tests

The possible release of chrysotile fibers, during maintenance or remediation of vinylasbestos, is a very controversial subject.

A study conducted by Paustenbach et al. [21], on four types of products containing asbestos embedded in a matrix, such as vinyl, reported a negligible concentration of airborne fibers. The research was carried out by simulating both removal and maintenance work through sanding or grinding. Their results showed an asbestos concentration below the limits indicated by the OSHA PEL [18] of 0.1 fiber/liter. They also found the presence of fibers below 5 microns in length in the air and incorporated in the matrix. These data reveal a non-hazardous risk to human health.

**Figure 11:**
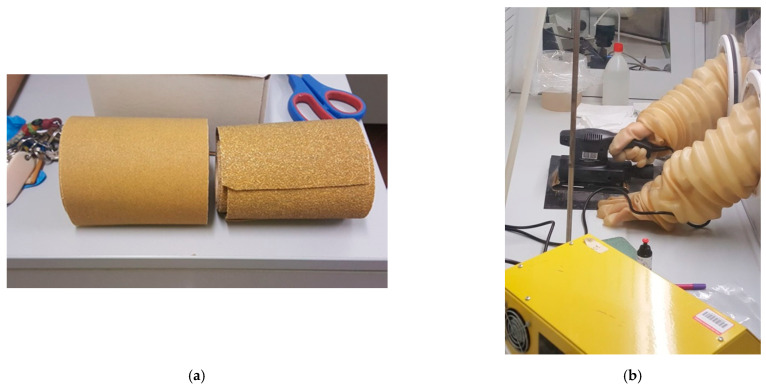
(**a**) Sandpaper used for the test. Left: fine sandpaper (dimensions: P120). Right: coarse sandpaper (dimensions: P40). (**b**) Performed mechanical abrasion test inside the confined hood.

In the present study, a strong mechanical abrasion of R and F vinyl tile samples was carried out using two types of sandpapers, one with a fine grain of P120 and one with a coarse grain of P40 (Figure 11). The abrasion time for each sample was 5 min.

Table 5 shows the quantity in grams of powder obtained during the abrasion test, and the quantity in grams of asbestos fibers contained in the powder.

**Figure 12:**
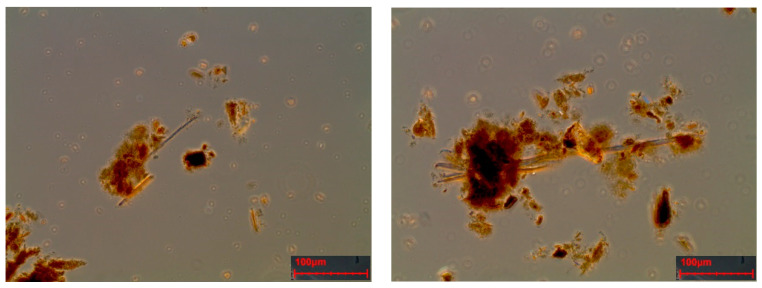
Phase-contrast microscope (PCOM) photos of the powder obtained by means of abrasion test. Chrysotile fiber with part of the vinyl glue remain attached.

The simulation tests performed on the two vinyl floor samples provided the airborne analysis for a quantitative evaluation of any release of asbestos fibers (free asbestos content). The release of free fibers into the powder, obtained from abrasion test was also quantitatively assessed (last column Table 5). The values obtained confirm, as shown in the D.M., that the percentage of chrysotile present in the vinyl flooring is in a range between 10% and 12% [3,4]. In Figure 12, two PCOM photos of the powder obtained by means of the abrasion testare reported; the presence of Chrysotile fibersis evident.

The evaluation of airborne samples was carried out considering only the breathable fibers, i.e., fibers with dimensions of length to diameter ratio > 3 (diameter < 3 microns and length > 5 microns). The evaluation of asbestos concentration in fibers/liter in the airborne samples was carried out considering Equation (1), suggested by the Italian M.D. 6/09/1994, Annex 2-B [4]. The quantitative determination of the concentrations of airborne asbestos fibers in indoor environments followed the scanning electron microscopy (SEM) method.

C = (n × π × d^2^)/(4 × N × A × V) (1)

where:

C = fiber/liter;

n = number of fibers counted;

d = the real powdered area of the membrane (mm^2^);

N = number of fields counted;

A = the area of the reading field (m^2^);

V = flow rate (m^3^).

## 3. Results and Discussion

### 3.1. Multigrip Plier Breakage Results

**Figure 13:**
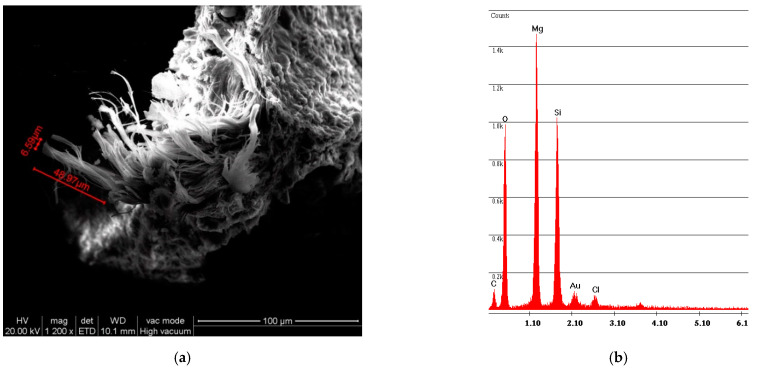
Sample F airborne sample of multigrip plier breakage simulation. (**a**) SEM photo; (**b**) spectrum with Mg and Si peaks indicating Chrysotile presence.

The simulation of the sampling was carried out by an operator to verify the presence of asbestos in the artefacts by mapping the areas containing asbestos; this simulation found a release of fibers that exceeds the limits of 2 fibers/liter only for sample R. The asbestos fiber concentration is C = 3.05 fibers/liter for sample R and C = 1.53 fibers/liter for sample F, indicating that sampling of asbestos-vinyl floors must be carried out avoiding asbestos fibers contamination for operators by adopting preventive and protective measures while operating in wet conditions [4]. Figure 12 and Figure 13 show two examples of SEM photos and spectrum of airborne samples.

**Figure 14:**
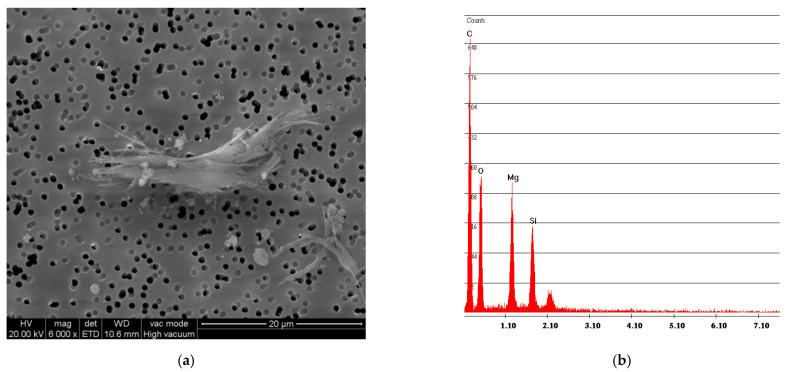
R airborne sample of multigrip plier breakage simulation. (**a**) SEM photo; (**b**) spectrum with Mg and Si peaks indicating Chrysotile presence.

Figure 13 shows that in sample F, many fibers remain incorporated in the adhesive matrix of the vinyl, while the fibers found for the sample R (Figure 14) are free fibers.

### 3.2. Impact and Drag with Crampons Results

**Figure 15:**
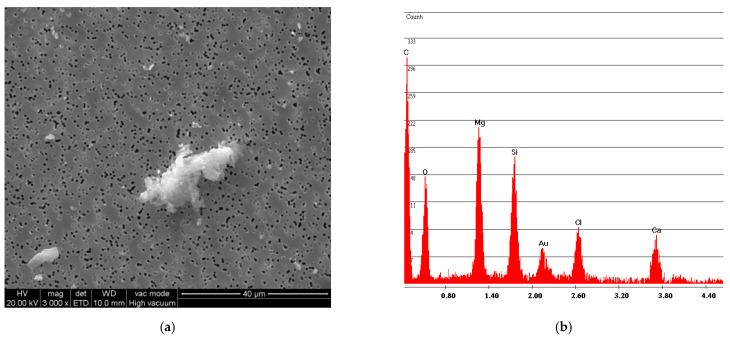
Sample R airborne sample of simulation for impact and drag with crampons. (**a**) SEM photo of incorporated asbestos fibers in the vinyl-adhesive matrix. (**b**) Spectrum with Mg and Si Chrysotile peaks and Cl and Ca adhesive matrix peaks.

**Figure 16:**
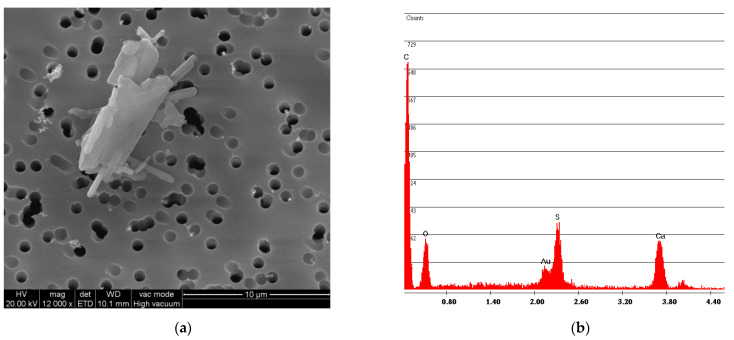
Sample F airborne sample of simulation for impact and drag with crampons. (**a**) SEM photo of part of the matrix without asbestos fibers. (**b**) Spectrum of the adhesive matrix.

This test was carried out with impacts and drag that simulate the trampling of the vinyl floor; this simulation did not produce fiber release. The test did not produce powder. The results obtained with Equation (1) are C = 0 fibers/liter for both samples. This confirms the indications given by the Italian Ministerial Decree 06/09/1994 [4], which states that there is a release of fibers only in the case of abrasion, perforation, brushing or if the vinyl floor is very deteriorated (Table 3). Figure 15 shows incorporated asbestos fibers in adhesive vinyl matrix. Figure 16 shows a piece of the adhesive matrix of the vinyl floor.

### 3.3. Abrasion Simulation Results

The abrasion test was carried out with two different abrasive papers. Both samples were abraded with fine-grained sandpaper and coarse-grained sandpaper that produced a fiber release above the limits [4].

**Figure 17:**
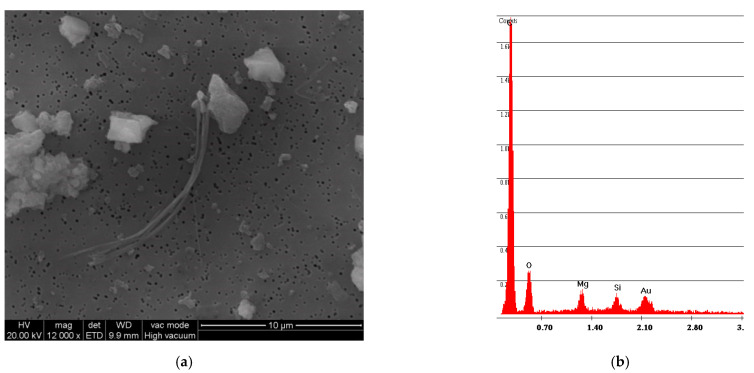
F airborne sample of abrasion simulation test with fine-grained sandpaper. (**a**) SEM photo of Chrysotile bundle. (**b**) Spectrum of Chrysotile with typical Mg and Si peaks.

**Figure 18:**
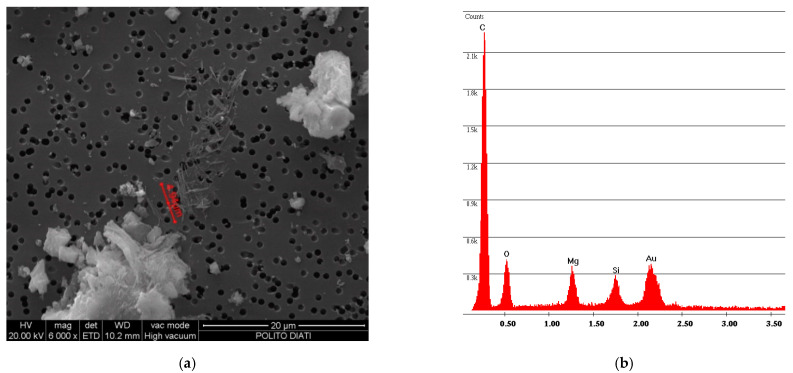
R airborne sample of abrasion simulation test with fine-grained sandpaper. (**a**) SEM photo of little Chrysotile free fibers;(**b**) spectrum of Chrysotile with typical Mg and Si peaks.

The results obtained for abrasion with fine-grained sandpaper are C = 12.97 fibers/liter for sample F and C = 24.42 fibers/liter for sample R. The abrasion of sample R produced more longer and free fibers compared to the ones of sample F. This particular aspect is shown in Figure 17 and Figure 18. The smaller fiber dimension of sample R had already been observed during the vinyl characterization test (Figure 6 and Figure 7).

**Figure 19:**
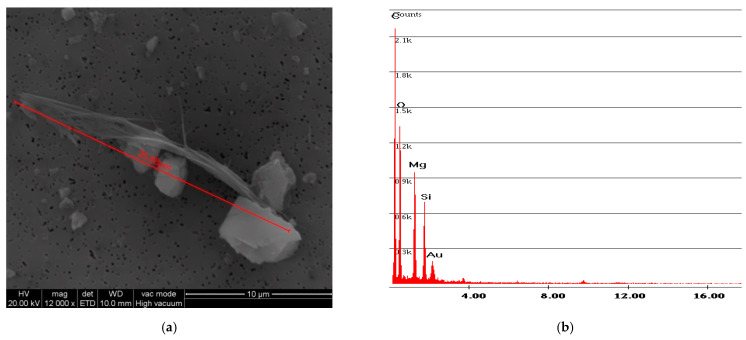
F airborne sample of abrasion simulation test with coarse-grained sandpaper. (**a**) SEM photo of Chrysotile bundle; (**b**) spectrum of Chrysotile with typical Mg and Si peaks.

**Figure 20:**
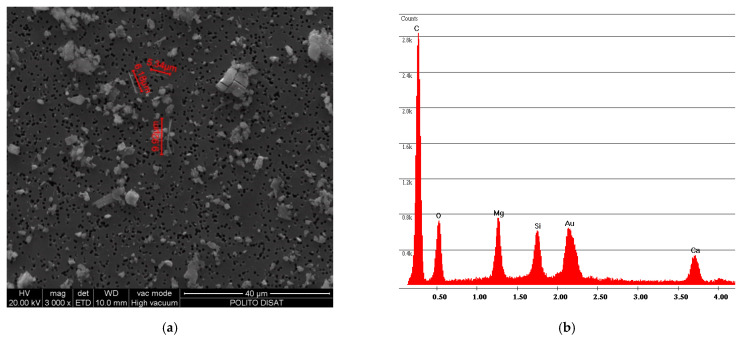
R airborne sample of abrasion simulation test with coarse-grained sandpaper. (**a**) SEM photo of Chrysotile free fiber bundle; (**b**) spectrum of Chrysotile with typical Mg and Si peaks.

The results obtained with abrasion simulation test using coarse-grained sandpaper were C = 9.16 fibers/liter for sample F and C = 7.63 fibers/liter for sample R. The free fibers obtained with coarse-grained sandpaper were larger than the free fibers obtained with fine-grained abrasive sandpaper for both samples. Furthermore, the same trend that was observed for fine-grained abrasive sandpaper can also be observed for coarse-grained abrasive sandpaper, i.e., the fiber sizes from sample F were larger than the fiber sizes of sample R (Figure 19 and Figure 20).

**Figure 21:**
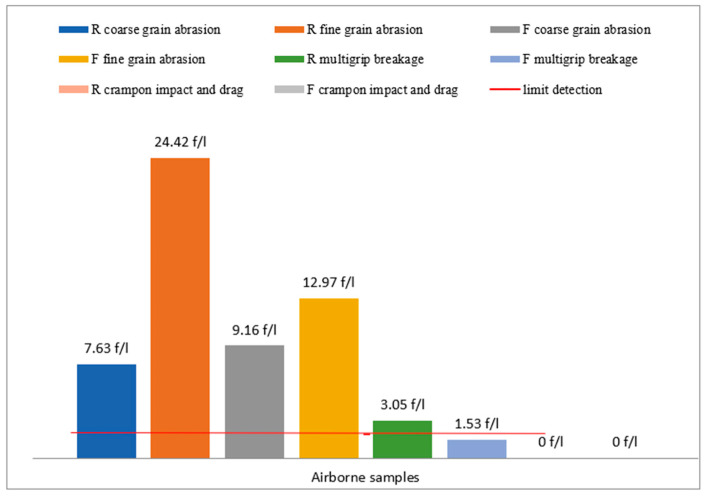
Histogram of the fiber/liter values calculated according to Ministerial Decree 06/09/1994 [4], for all tests carried out on samples R and F. The horizontal red line indicates the limit detection value.

Figure 21 shows a representation of all simulation tests on samples R and F. The red line represents the limit detection value according to Italian Ministerial Decree 06/09/1994 [4]. It can be observed that, in general, the R sample shows worse results than the vinyl sample F, due to its rigidity and ageing comparing to vinyl floor F.

The only test that did not produce liberation of fibers was the crampon impact and drag. The other tests released fibers, in particular the abrasion simulation test. Lange [22] investigated the release of the asbestos fibers during the removal of floor tiles using scraping and lifting with ice scrapers with little or no water applied. He showed that the release of fibers, in this case, was low and that the probability of exposure is minimal. This result can be assimilated to the two drag and impact simulations with the crampon and multiplier breakage, which reported very low fiber release levels, even though they were carried out dry. However, the difference between a good, flexible floor and a bad, rigid floor must be taken into consideration during the removal operation, because the fiber release for rigid and poor-conditioned floor tile is higher.

Crossman [23] states that the risk of human exposure from asbestos-containing material is directly related to the condition of the material, the custody/maintenance activities and the methods used to reduce potential exposure to workers. The deterioration of vinyl floors during polishing, removal and routine walking, due to the friction generated by these activities, causes the release of fibers. Moreover, the level of fiber release varies with the aggressiveness of these procedures. This aspect was found in this study by observing the difference in abrasion fiber release compared to other simulations tests.

## 4. Conclusions

As defined by the Italian Ministerial Decree 6/9/94 [4], the release of fibers from a vinyl-containing asbestos can only occur if it is abraded, perforated or cut, but not during its normal use, even if the vinyl floor is in place for more than 30 years. Furthermore, no clear methodologies are suggested for removal due to the installation of another floor or for confinement with a new flooring complete with maintenance.

Analyzing one case at a time, we can provide the following suggestions:

The impact and drag simulation with crampons mimics the passage of people with shoes and the effect of a small stone fragment stuck in the sole of rubber shoes, or any case the normal walking routine. The simulation led to a non-release of fibers for both types of flooring, rigid and flexible. This confirms what is indicated by legislation, that there is no release of fibers in the normal use of vinyl, due to their incorporation within the matrix, independent of ageing.

The multigrip breakage simulation of vinyl tiles, on the other hand, reports a limited release of fibers that, especially in the case of rigid, old and worn floors, can exceed the limits defined by the regulations. Therefore, when sampling or removing the vinyl flooring, if it is broken, it can cause the release of asbestos fibers. The suggestion, in this case, is, therefore, to carry out this operation in wet safety, as has also been suggested by Kominsky et al. [13]; they state that even for normal maintenance operations, especially stripping, removal should be carried out in wet conditions and using PPE (personal protective equipment). After the removal of the floor tiles, the airborne analysis must be carried out to verify that the environment is clean from fibers. In this case, the wear and the condition of vinyl floor tiles are important for the release of fibers.

The simulation with abrasion with the two different types of abrasive sandpaper produced a remarkable release of fibers in all cases and for both floors. This once again confirms what is stated in the legislation: the abrasion, perforation or cutting could release asbestos fibers. Operations such as dragging furniture or heavy materials must be carried out with care to avoid causing abrasion of the flooring. The effect of grains stuck to the soles of shoes can also cause abrasion and, therefore, the release of asbestos fibers.

During the vinyl floor removal, part of the flooring often remains attached to the sub-floor. The cleaning of this portion of vinylasbestos, probably with glue, should be carried out with great care under wet conditions to prevent a high release of fibers. The use of solvents for the removal of the underlying glue instead of abrasion must be carefully studied, as previous studies have shown. The use of solvents can break the bonds of the glue matrix with part of asbestos fibers, thus freeing them [1].

## Figures and Tables

**Table 1:** Components and percentage of typical vinylasbestos for US patents 2558378A of Petry [2].

Components		Parts by Weight: Range	Parts by Weight: Optimal Proportion	Particle Size Dimension (Mesh)
polymer		16–38	23	
plasticizer		7–18	12	
filler	-pigments	50–75	65	<325
	-fibrous fillers such as wood flour dispersed in plastic			<50
	-asbestos			<10

**Table 2:** Components and percentage of typical vinylasbestos according to US patents: US3523095A of Laurito and Wheeler [3].

Components	Percentages (%)
homopolymer vinyl chloride and copolymer	15–18
primary plasticizer	5.3–6.5
stabilizer	0.6–0.8
asbestos	11–25
limestone	48–63
pigments	2–4

**Table 3:** Main types of asbestos-containing materials and their approximate fiber potential release (from Article 1, Annex: Standards and Technical Methods for Risk Assessment, Control, Maintenance and Remediation of Materials Containing Asbestos Present in Building Structure, Table 1 of M.D. 6/9/94 [4].

Asbestos-Containing Materials (ACMs)	Note	Friability
Sprayed coatings, lagging and insulations	Up to about 85% of asbestos (amosite, crocidolite) mainly amosite sprayed on steel bearing structures or other surfaces as thermal-acoustic insulators	High friability
Insulating pipes and boilers lagging	For pipe coatings, all types of asbestos, about 6–10%, usually mixed with calcium silicates. In cloths, felts and padding, in general, 100% of asbestos	High release potential in case of coating covering layer absent, not uniformed or deteriorated
Cardboard, paper and similar	Generally, only 100% chrysotile	Subjected to wear and tear due to their poorly compact structure
Tapes, cords and cloths	In the past, all types of asbestos were used. Later, only chrysotile	Possibility of fiber release when large quantities of material are stored
Asbestos cement products	Currently 10–15% of asbestos, usually chrysotile. Crocidolite and amosite can be found in some types of pipes and slabs	Possibility of fiber release when they are abraded, sawn, perforated or brushed, or if deteriorated
Bituminous and tar products, paper-backed vinyl tiles, vinyl floor tiles, PVC and plastic goods, coatings and paints, mastics, sealants and plaster	From 0.5 to 2% for mastics, sealants, adhesives, at 10–25% for floors and vinyl tiles	Unlikely release of fibers during normal use. Possibility of release of fibers if cut, abraded or perforated

**Table 4:** Density of two vinyl samples F (green and flexible) and R (black and rigid).

Samples	Density (g/cm^3^)
F	1.82
R	1.75

**Table 5:** Quantity of powder produced during abrasion test and quantity of asbestos fibers contained in the powder.

Simulation Test	Vinyl Floor Tile Sample	Powder Produced from Abrasion (g)	Release of Asbestos Fibers (g)
Abrasion coarse-grain sandpaper	F	4.4	0.4
	R	6.2	0.7
Abrasion fine-grain sandpaper	F	4	0.4
	R	5.3	0.6
